# 

**Published:** 1906-07-15

**Authors:** 


					﻿Vol. LX.	JULY 15, 1906.	No. 7.
tiie I
DENTAL REGISTER
EDITED BY
NELVILLE S. HOFF, D.D.S.,
ANN ARBOR, MICH.
PRICE, ONE DOLLAR A YEAR IN ADVANCE.
Published Monthly by
SAMVEL A. CROCKER & CO.,
Ohio Dental and Surgical Depot.
35 to 39 W. 5th St., Cincinnati, 0.
Entered at the Postoffice at Cincinnati, O., as second-class mail matter.
				

